# Human umbilical cord mesenchymal stem cells improve the reserve function of perimenopausal ovary via a paracrine mechanism

**DOI:** 10.1186/s13287-017-0514-5

**Published:** 2017-03-09

**Authors:** Jia Li, QiuXian Mao, JingJun He, HaoQing She, Zhi Zhang, ChunYan Yin

**Affiliations:** 1 Department of Obstetrics and Gynecology, Graduate College, Guangdong Medical University, Zhanjiang, Guangdong 524023 China; 2 Department of Obstetrics and Gynecology, Guangdong No.2 Provincial People’s Hospital, NO.466 Xingangdong Road, Guangzhou, 510317 China; 3 Department of Physical Examination, Guangdong No.2 Provincial People’s Hospital, NO.466 Xingangdong Road, Guangzhou, 510317 China; 4 Department of Obstetrics and Gynecology, Medical College, NanHua University, Hengyang, Hunan 421001 China; 5 Department of Laboratory Medicine, Guangdong No.2 Provincial People’s Hospital, NO.466 Xingangdong Road, Guangzhou, 510317 China

**Keywords:** hUCMSCs, Perimenopausal, Ovarian reserve function, HGF, VEGF, IGF-1, Paracrine

## Abstract

**Background:**

Human umbilical cord mesenchymal stem cells (hUCMSCs) are a type of pluripotent stem cell which are isolated from the umbilical cord of newborns. hUCMSCs have great therapeutic potential. We designed this experimental study in order to investigate whether the transplantation of hUCMSCs can improve the ovarian reserve function of perimenopausal rats and delay ovarian senescence.

**Method:**

We selected naturally aging rats confirmed by vaginal smears as models of perimenopausal rats, divided into the control group and the treatment group, and selected young fertile female rats as normal controls. hUCMSCs were transplanted into rats of the treatment group through tail veins. Enzyme-linked immunosorbent assay (ELISA) detected serum levels of sex hormones, H&E staining showed ovarian tissue structure and allowed follicle counting, immunohistochemistry and western blot analysis revealed ovarian expression of hepatocyte growth factor (HGF), vascular endothelial cell growth factor (VEGF), and insulin-like growth factor-1 (IGF-1), polymerase chain reaction (PCR) and western blot analysis revealed hUCMSCs expression of HGF, VEGF, and IGF-1.

**Results:**

At time points of 14, 21, and 28 days after hUCMSCs transplantation, estradiol (E_2_) and anti-Müllerian hormone (AMH) increased while follicle-stimulating hormone (FSH) decreased; ovarian structure improved and follicle number increased; ovarian expression of HGF, VEGF, and IGF-1 protein elevated significantly. Meanwhile, PCR and western blot analysis indicated hUCMSCs have the capacity of secreting HGF, VEGF, and IGF-1 cytokines.

**Conclusions:**

Our results suggest that hUCMSCs can promote ovarian expression of HGF, VEGF, and IGF-1 through secreting those cytokines, resulting in improving ovarian reserve function and withstanding ovarian senescence.

## Background

Menopause is the permanent termination of menstruation because of loss of ovarian follicular activity. Perimenopause means the coming of menopause, which manifests itself as menstrual irregularity and vasomotor symptoms, it ends in the 12 months after the final menstrual period [1]. Most women experience perimenopause classically between the ages of 45 and 55 years [2]. Perimenopause can last for several years or even decades, and brings many perimenopausal symptoms such as hot flushes, vaginal atrophy, osteoporosis, depression, etc. [3, 4]. Currently, hormone replacement therapy (HRT), symptomatic supporting treatment, and treatment with phytoestrogens or herbal remedies are the three main treatments of perimenopausal symptoms. However, HRT may have a long-term effect on increasing the risk of breast cancer, endometrial cancer, and ovarian cancer; symptomatic supporting treatment cures the symptoms, not the disease; and treatment with phytoestrogens or herbal remedies lacks data on the mechanism and long-term safety [1, 4]. The delay of childbearing as an important social change has led to an increasing number of women desiring late menopause, and the improvement of the quality of life means women want to avoid the trouble of perimenopausal symptoms and to slow down the rapidity of ovarian aging. In addition, follicles have limited numbers. Several million non-growing follicles (NGFs) are established by the ovary at around 5 months of gestational age, then decline to approximately 1000 when the menopause starts, and are finally exhausted through atresia and apoptosis after 12–14 years of menopause [5, 6]. Therefore, how to make the best use of NGFs, delay ovarian senescence, and cure perimenopausal syndrome fundamentally are a serious problem in today’s society.

Human mesenchymal stem cells (MSCs) have attracted great interest recently, due to their huge therapeutic potential. Human umbilical cord mesenchymal stem cells (hUCMSCs) are obtained directly from the Wharton’s jelly of a human umbilical cord, and are also called human Wharton’s jelly mesenchymal stem cells (WJ-MSCs). Fewer ethical issues, being obtained painlessly from abandoned umbilical cord, and being hypoimmunogenic are the prominent advantages of hUCMSCs compared to other sources of MSCs [7]. The ability to modulate immune responses makes hUCMSCs an important stem cell source for allogeneic transplantation therapy without immunological rejection [8]. Troyer and Weiss [9] concluded that there was no evidence for direct immunological rejection of undifferentiated hUCMSCs in vivo and they would be accepted well in allogeneic transplantation. Besides, Gong et al. [10] discovered that there were no signs of immunologic response and no evidence in the dosage escalation and frequencies of hUCMSCs used in patients. Furthermore, hUCMSCs have multipotent stem cell characteristics, which can differentiate into multiple lineages under different differentiation conditions [11]. Some studies also showed [12, 13] that hUCMSCs differentiated into oocyte-like structures and expressed both mRNA and protein of germ cell-specific markers. hUCMSCs increased the proliferation of damaged human endometrial stromal cells (ESCs) and decreased the apoptosis percentage significantly when cultured with them. Yang et al. [14] suggested that hUCMSCs may restore endometrial damage through secreting vascular endothelial growth factor (VEGF) and anti-apoptosis. Zhu et al. [15] confirmed that hUCMSCs transplantation could restore ovaries damaged by chemotherapy in rats. This experiment was lately verified by Song et al. [16]. They all proposed that the improved ovarian function in premature ovarian failure (POF) rat model was more likely due to the cytokines produced by hUCMSCs via a paracrine mechanism rather than directly differentiating to germ cells.

Early follicle-stimulating hormone (FSH) was a main endocrine feature of perimenopause, reported by Sherman and Korenman in 1975 for the first time [17], and has been used since the 1990s as a biomarker of reproductive potential [18]. Mean FSH levels between the earliest menopausal phase of every definition showed statistically significant differences, and serum estradiol (E_2_) levels decrease and FSH levels increase with growing age in midlife women [19]. Therefore, Gracia et al. [20] suggested that delicate changes in blood may be helpful in recognizing the earliest hormonal changes during the transition to menopause. However, the increase of FSH levels only happens around 10 years before the menopause in which infertility perhaps starts. Thus, a markedly raised FSH is considered a relatively late predictor for menopausal transition [21]. Anti-Müllerian hormone (AMH), as a member of the transforming growth factor beta (TGF-β) family, can affect the transition from NGFs to growing follicles. Hence it is viewed currently as the best available predictor of ovarian reserve [22]. AMH levels remain relatively stable over the menstrual cycle; therefore, measurement does not need to be conducted on a specific cycle day. In such a way, AMH has the advantage over FSH [18]. Individual AMH serum level reflects the size of the antral follicles pool, representing the quantity of NGFs accurately [23]. Since AMH production decrease is in accord with the age-related decline in the number of antral follicles, AMH levels can be used as a label for ovarian aging [24–26].

Based on the precedents discussed above, we designed this experiment to investigate the therapeutic potential of hUCMSCs using perimenopausal rats. We established the perimenopausal sample by selecting naturally aging rats, confirmed by vaginal smears and the level of serum hormone. The sample is in accordance with the status of perimenopausal women. In addition, we chose serum level of E_2_, AMH, and FSH as the evaluation index of ovarian reserve function. Our study aimed to identify the potential of hUCMSCs in perimenopausal treatment and the mechanism involved.

## Methods

### Animals

The naturally aging female Sprague-Dawley rats (SPF class, weight 410–450 g, 12–14 months old) and young female Sprague-Dawley rats with fertility (SPF class, weight 280–320 g, 3–5 months old) were provided by Guangdong Medical Laboratory Animal Center (Foshan City, China). They were bred at a temperature of 30 ± 2 °C with a 12-hour light/dark cycle. Vaginal smears of rats were taken to determine estrous cycle at 11:00 am daily. Only aging rats with disorganized estrous cycles and young rats with normal estrous cycles were chosen [27, 28]. Before the experiment, 1 ml of blood was gathered from each rat’s orbital during dioestrus. We centrifuged blood samples for 10 minutes at 2000 rpm after 45 minutes’ standing and reserved the upper serum at -80 °C. Initial levels of rat E_2_, FSH, and AMH were accessed for each serum sample. Aging rats were distributed into the control group and the treatment group (n = 15 per group) randomly. Young rats were set as the normal control group (n = 15).

### Identification of hUCMSCs phenotype

The P1 generation cell lines of hUCMSCs were obtained from ChongQing HuaYa Stemcell Technology Corporation (ChongQing, China). hUCMSCs were isolated from human umbilical cords of newborns. Maternal blood passed the etiological examination, proving that they were not infected by treponema pallidum, hepatitis B virus, and human immunodeficiency virus. Flow cytometry was used to identify the phenotype of hUCMSCs. CD73(eBioscience, Inc., San Diego, CA, USA, 12-0739-41), CD90 (eBioscience, 11-0909-41), CD105 (eBioscience, 12-1057-41), CD14 (eBioscience, 11-0149-41), CD34 (eBioscience, 12-0349-41), CD45 (eBioscience, 11-0459-41), CD79a (eBioscience, 12-0792-41) and HLA-DR (eBioscience, 11-9952-41) monoclonal antibodies were used for detection. Mouse IgG monoclonal antibody was used as negative control. Cells at a concentration of 2 × 10^6^ cells/ml were incubated with 5 ul antibodies (for each) at 4 °C for 30 minutes and were analyzed by flow cytometer (Beckman Coulter, Brea, CA, USA, MoFlo Astrios EQ).

### Treatment

hUCMSCs were cultured in complete medium (DEME/F12 with 10% FBS and 1% penicillin-streptomycin) until they fulfilled the quantity. And then the P3 generation of hUCMSCs was suspended in phosphate-buffered saline (PBS) at a concentration of 1 × 10^6^ cells/ml. The hUCMSCs suspension was injected into the treatment group via the tail vein (1 ml per rat). After 48 hours, the treatment group was again injected with 1 ml hUCMSCs suspension. To avoid cell clusters causing rats thrombosis, cell suspension should be adequately scattered before injection. The control group was injected with 1 ml PBS per rat via the tail vein at the same time. No treatment was administered to the normal control group. At 14, 21, and 28 days after the final cell transplantation, five rats from each group were selected randomly and euthanized. Blood was taken from orbits and ovaries were removed for analysis.

### Measurement of rats’ E_2_, FSH, and AMH levels

Blood samples were standing for 45 minutes and centrifuged for 10 minutes at 2000 rpm. The supernatant serum was collected and reserved at -80 °C. Levels of sera E_2_, FSH, and AMH were measured by enzyme-linked immunosorbent assay (ELISA) kit E_2_ (Cusabio Biotech Co., Ltd, Wuhan, China, CSB-E05110r), FSH (CUSABIO, CSB-E06869r), and AMH (CUSABIO, CSB-E11162r).

### H&E staining showed ovarian tissue structure and allowed follicle counting

Left ovaries were removed for fixation in 10% formalin to prepare the paraffin sections. Each section was 3 um thick. Sections were stained with hematoxylin and eosin (H&E) and observed under a light microscope. The number of follicles at different stages was counted according to definition. Primordial follicles were defined as an oocyte surrounded by a single fusiform granule cell. Primary follicles were an oocyte surrounded by a single layer of cuboidal granulosa cells. Secondary follicles were surrounded by six to eight layers of cuboidal granulosa cells, with no visible antrum. Antral possessed a clearly defined antral space [29].

### PCR analysis hUCMSCs HGF, VEGF, and IGF-1 mRNA expression

The P3 generation of hUCMSCs was cultured in culture dishes until they grew to 90% confluence. After that, culture media were abandoned and cells were washed in PBS three times. The total RNA of the cells was extracted using E.Z.N.A.Total RNA Kit I (Omega Bio-tech, Inc., Norcross, GA, USA, R6834-01). Before RNA was transcribed, DNase I (Beyotime, Shanghai, China, D7076) had been added to remove the genome DNA. Ultraviolet spectrophotometry was used to determinate the concentration of total RNA. RNA was transcribed reservedly to cDNA using PrimeScript™ 1st Strand cDNA Synthesis Kit (Takara Bio Inc., Kyoto, Japan, 6110A), and cDNA was amplified by Premix Taq™ version 2.0 plus dye kit (Takara Bio USA Inc., Mountain View, CA, USA, RR901Q). Distilled water was used to replace cDNA as the blank control of quality when amplified. Human hepatocyte growth factor (HGF), VEGF and insulin-like growth factor-1 (IGF-1) primer (both from Sangon Biotech, Shanghai, China) was designed according to GeneBank, primer sequences are as below. HGF: forward (5′-GCAATTAAAACATGCGCTGA-3′), reverse (5′-TGGAATTTGGGAGCAGTAGC-3′), overall length is 269 bp; VEGF: forward (5′-GGGGAGGAGGAAGAAGAGAA-3′), reverse (5′-GTGGAGGTAGAGCAGCAAGG-3′), overall length is 315 bp; IGF-1: forward (5′-AGGGTATGGCTCCAGCAGTC-3′), reverse (5′-GAGGGGTGCGCAATACATCT-3′), overall length is 106 bp. 5 ul purpose gene amplification product was taken for electrophoresis on 2% agarose gel. Observation under the gel imaging analysis system was conducted, with photographs taken.

### Western blot analysis of hUCMSCs expression of HGF, VEGF, and IGF-1

The P3 generation of hUCMSCs was cultured in culture dishes until they grew to 90% confluence. After that, the culture media was abandoned and cells were washed in PBS three times. The total protein was extracted from the cells using a total protein extraction kit (Nanjing Keygen Biotech Co. Ltd, Nanjing, China, KGP250) and the protein content was measured by BCA protein quantitation assay kit (Keygen Biotech, KGP904). Protein was taken according to the quantitation for electrophoresis on 12% SDS-PAGE gel. We used 5% skimmed milk with TBST and closed PVDF membrane after transmembrane. The primary antibodies used HGF (1:1500 dilution; ab83760; Abcam, Cambridge, MA, USA), VEGF (1:1200 dilution; ab46154; Abcam), IGF-1 (1:600 dilution; ab176523; Abcam) for the reaction at 4 °C overnight. The second antibodies used HRP-conjugated Affinipure Goat Anti-Mouse IgG (H + L) or HRP-conjugated Affinipure Goat Anti-rabbit IgG (H + L) (1:8000 dilution; SA00001-1 or SA00001-2; Proteintech, Wuhan, China) for the reaction at room temperature for 1 hour. Chemiluminescence used the Tanon 5200 analysis system (Tanon Science & Technology Co Ltd., Shanghai, China).

### Immunohistochemistry analysis of ovarian expression of HGF, VEGF, and IGF-1

The ovary sections were immunohistochemically stained for HGF, VEGF, and IGF-1 antibodies in order to explore the cytokine expression in ovaries after hUCMSCs transplantation. Antigen was retrieved by microwave antigen retrieval in sodium citrate buffer (pH6.0). Immunohistochemistry (IHC) assay used the S-P method (ZSGB-Bio, Beijing, China, SP-9000-D). The primary antibodies HGF (1:300 dilution; ab83760; Abcam), VEGF (1:300 dilution; ab46154; Abcam) and IGF-1 (1:50 dilution; ab176523; Abcam) were used for the reaction at 4 °C overnight. The primary antibody was replaced by PBS as the negative control. DAB kit (ZSGB-Bio, ZLI-9017) was used for staining. All sections were observed and semiquantitatively analyzed under a light microscope by two pathologists independently. The immunoreactive score (IRS) is according to their staining intensity and positive range at high magnification (×400). Staining intensity was divided into four levels (negative, weak, intermediate, strong), and given 0–3 scores respectively. The positive range was divided into five levels and given 0–4 scores respectively according to the positive cells’ percentage of the microscopic view (0–10%, 11–25%, 26–50%, 51–75%, or 76–100%). Ten view-zones were chosen for each section, which avoided overlaps on observation. The score of staining intensity multiplying the score of the positive range decides the score of a view zone and the average score of ten view zones determines the score of a staining section.

### Western blot analysis of ovarian expression of HGF, VEGF, and IGF-1

Right ovaries were washed in saline and stored at -80 °C. Each ovary’s total protein was extracted for the expression of HGF, VEGF, and IGF-1 assay by western blotting. The experiment method was the same as the cells’ western blot assay method. The Tanon 5200 Chemiluminescence Imaging System was used to analyze image intensity. The standardization ratio of target protein intensity to GAPDH (1:8000 dilution; 3777R-100; BioVision, Inc., Milpitas, CA, USA) intensity was viewed as the reference index of expression quantity.

### Statistical analysis

All data were analyzed using IBM Statistical Program for Social Sciences 20.0 (IBM Corp., Armonk, NY, USA). The results were shown as the mean ± standard deviation. Data from the same group at different time points and data from multiple groups at the same time point were analyzed by one-way ANOVA test. The contrast between two groups used Students’ *t* test. *P* values were considered significant when less than 0.05.

## Results

### Phenotype characterization of hUCMSCs

**Fig. 1:**
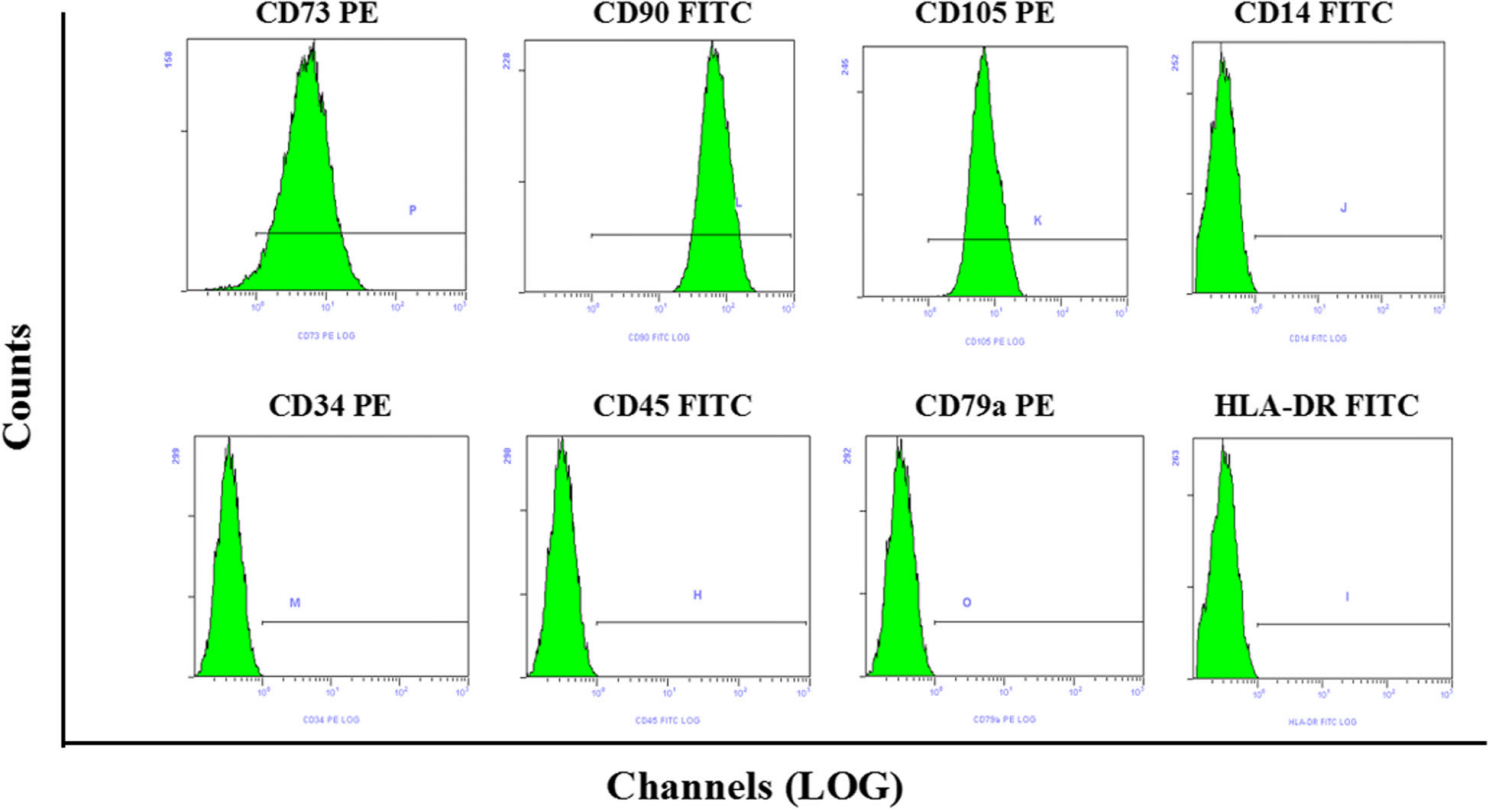
Flow cytometry analysis of phenotype characterization of hUCMSCs. Phenotype of CD73, CD90, CD105, CD14, CD34, CD45, CD79a and HLA-DR of hUCMSCs was detected by flow cytometry. Intensity ≥ 95% represented strong expression while ≤2% represented low or no expression

Flow cytometry test results showed that hUCMSCs express the specific markers of mesenchymal cell such as CD73, CD90, CD105 (≥95%) while they do not express CD14, CD34, CD45, CD79a and HLA-DR (≤2%) (Fig. 1).

### hUCMSCs transplantation does not cause rats’ graft rejection

There was no difference between the three groups of rats in their mental status, diet, hair color, and activity after transplantation. Moreover, there were no deaths, bleeding, hemiplegia, convulsions, and other graft rejection reactions in the treatment group. This implies that hUCMSCs transplantation is safe for Sprague-Dawley rats.

### hUCMSCs transplantation improves rats’ ovarian reserve function

**Fig. 2:**
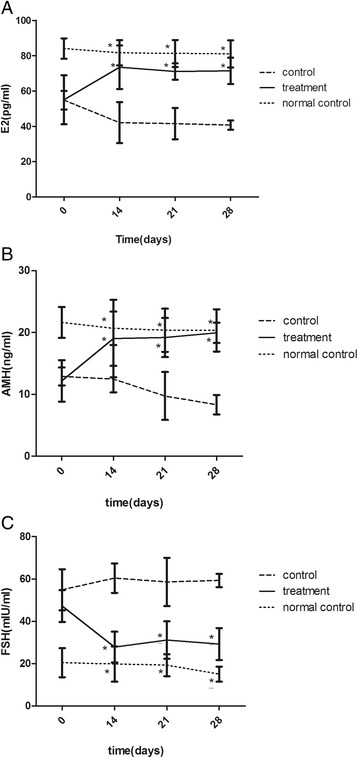
Serum hormone level analysis. Levels of E_2_ (**a**), AMH (**b**), and FSH (**c**) were measured by ELISA at 0 (when the perimenopausal model was established), 14, 21, and 28 days after hUCMSCs transplantation. ^*^ *P* < 0.05 vs control group. *AMH* anti-Müllerian hormone, *E*_*2*_ estradiol, *FSH* follicle-stimulating hormone

Before hUCMSCs transplantation, rats’ serum E_2_ and AMH level of the control group and the treatment group significantly decreased, while FSH increased compared to the normal control group. On the other hand, there was no significant difference between the two perimenopausal groups. After hUCMSCs transplantation, however, rats’ serum E_2_ and AMH of the treatment group increased and FSH decreased. This tendency has clear differences compared with the control group (Fig. 2; *P* < 0.05), yet has no significant difference between each time period of 14, 21, and 28 days after hUCMSCs treatment. Rats’ sera hormone level of the normal control group has no change at each time point.

### hUCMSCs transplantation improves rats’ ovary structure and follicle counting

**Fig. 3:**
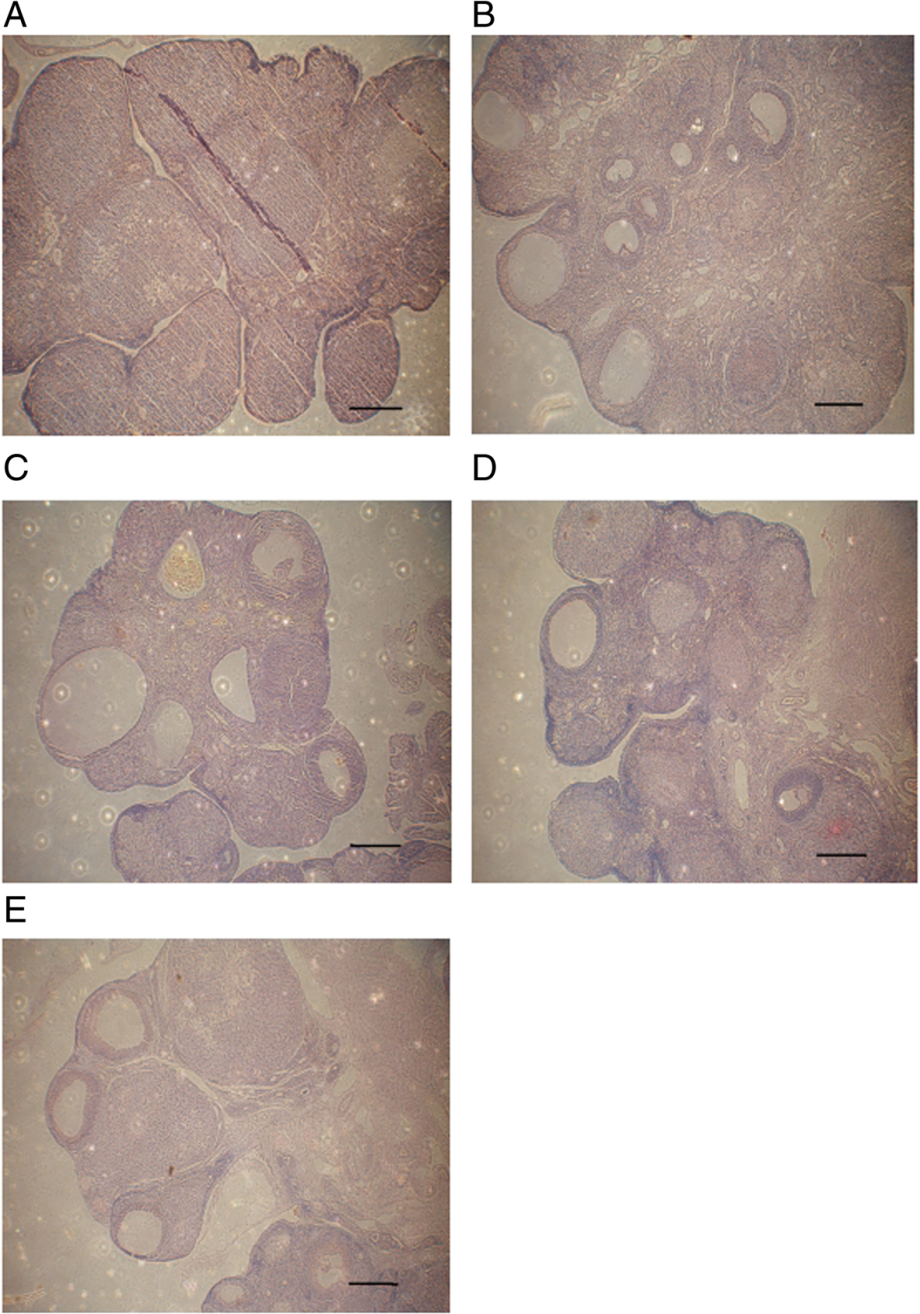
H&E staining analysis of ovarian structures. **a** Control group. **b** Treatment group at 14 days after hUCMSCs transplantation. **c** Treatment group at 21 days after hUCMSCs transplantation. **d** Treatment group at 28 days after hUCMSCs transplantation. **e** Normal control group. Scale bar = 500 um

Primordial, primary, secondary, and antral follicles were classified and counted according to the previous description and definition (Tables 1, 2, and 3). In the normal control group, histomorphology of normal ovaries were observed (Fig. 3e). Conversely, in the control group, we found that ovarian stromal showed densification, the number of follicles at different periods were decreased (Fig. 3a). Expectedly, in the treatment group, developmental condition of follicles tended to improve, the number of ovarian follicles was increased (Fig.3b, c, d). The forms of rats’ ovarian tissue at different time points after transplantation were similar in the treatment group.

**Table 1:** The number of primordial, primary, secondary, and antral follicles at 14 days after transplantation

Group	Primordial	Primary	Secondary	Antral
Control	20.20 ± 2.86	7.00 ± 1.00	8.60 ± 2.07	1.40 ± 0.89
Treatment	28.40 ± 4.15^*^	13.80 ± 3.56^*^	12.60 ± 3.91	3.80 ± 1.48^*^
Normal control	34.60 ± 6.07^*^	16.00 ± 5.05^*^	14.40 ± 4.22^*^	8.20 ± 1.79^*^
*F* value	12.562	8.423	3.535	28.774
*P* value	0.001	0.005	0.062	<0.001

Data are shown as mean ± SD. (n = 5)

^*^ *P* < 0.05 vs. control group

**Table 2:** The number of primordial, primary, secondary, and antral follicles at 21 days after transplantation

Group	Primordial	Primary	Secondary	Antral
Control	17.40 ± 2.70	7.60 ± 1.52	5.80 ± 1.30	2.00 ± 0.71
treatment	26.80 ± 2.77^*^	12.00 ± 1.22^*^	10.60 ± 2.70^*^	4.60 ± 1.14^*^
Normal control	33.00 ± 7.75^*^	16.40 ± 4.62^*^	14.60 ± 4.04^*^	8.40 ± 1.67^*^
*F* value	12.339	11.570	11.510	33.783
*P* value	0.001	0.002	0.002	<0.001

Data are shown as mean ± SD. (n = 5)

^*^ *P* < 0.05 vs. control group

**Table 3:** The number of primordial, primary, secondary, and antral follicles at 28 days after transplantation

Group	Primordial	Primary	Secondary	Antral
Control	21.80 ± 7.22	8.80 ± 3.90	6.80 ± 1.64	2.80 ± 0.84
Treatment	27.80 ± 2.77	13.80 ± 2.77^*^	10.60 ± 1.82^*^	5.00 ± 1.00^*^
Normal control	35.00 ± 5.57^*^	17.00 ± 3.74^*^	15.20 ± 3.77^*^	8.20 ± 1.92^*^
*F* value	6.943	13.139	7.208	20.481
*P* value	0.010	0.001	0.009	<0.001

Data are shown as mean ± SD. (n = 5)

^*^ *P* < 0.05 vs. control group

### hUCMSCs can secrete HGF,VEGF, and IGF-1

**Fig. 4:**
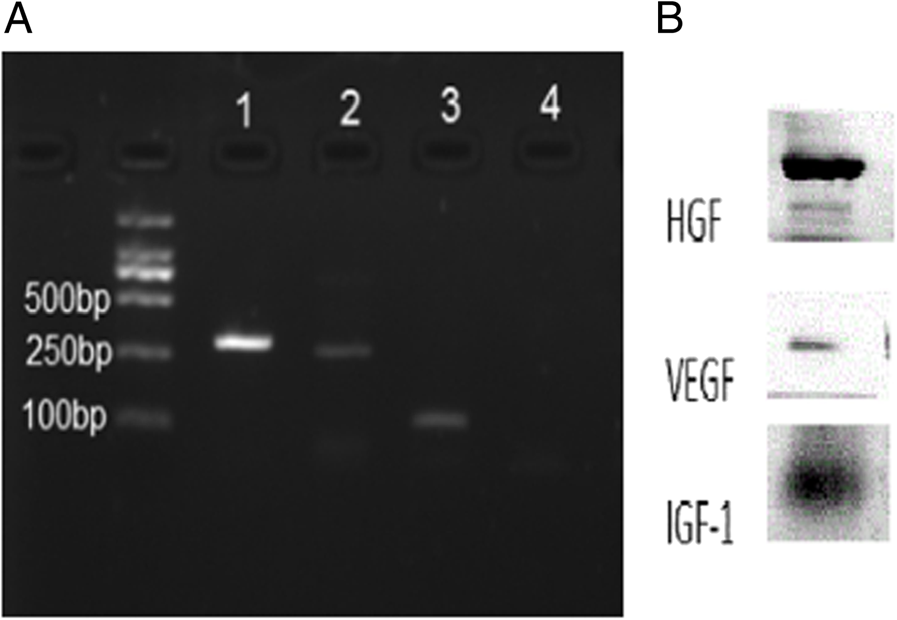
Analysis of hUCMSCs mRNA expression and protein expression. **a** Gel imaging analysis of hUCMSCs mRNA expression. *Lanes 1–4* were respectively for HGF, VEGF, IGF-1 mRNA, and blank control of quality. **b** Western blot images of HGF, VEGF, and IGF-1 protein expression of hUCMSCs. *HGF* hepatocyte growth factor, *IGF-1* insulin-like growth factor-1, *VEGF* vascular endothelial cell growth factor

Polymerase chain reaction (PCR) results suggested that hUCMSCs expressed HGF, VEGF, and IGF-1 mRNA (Fig. 4a). Meanwhile, western blot results suggested that hUCMSCs expressed HGF, VEGF, and IGF-1 protein (Fig. 4b). Western blot results were in accordance with PCR results, proving that hUCMSCs can secrete HGF, VEGF, and IGF-1 cytokines.

### HGF, VEGF, and IGF-1 expression in rats’ ovaries were improved after hUCMSCs transplantation

**Fig. 5:**
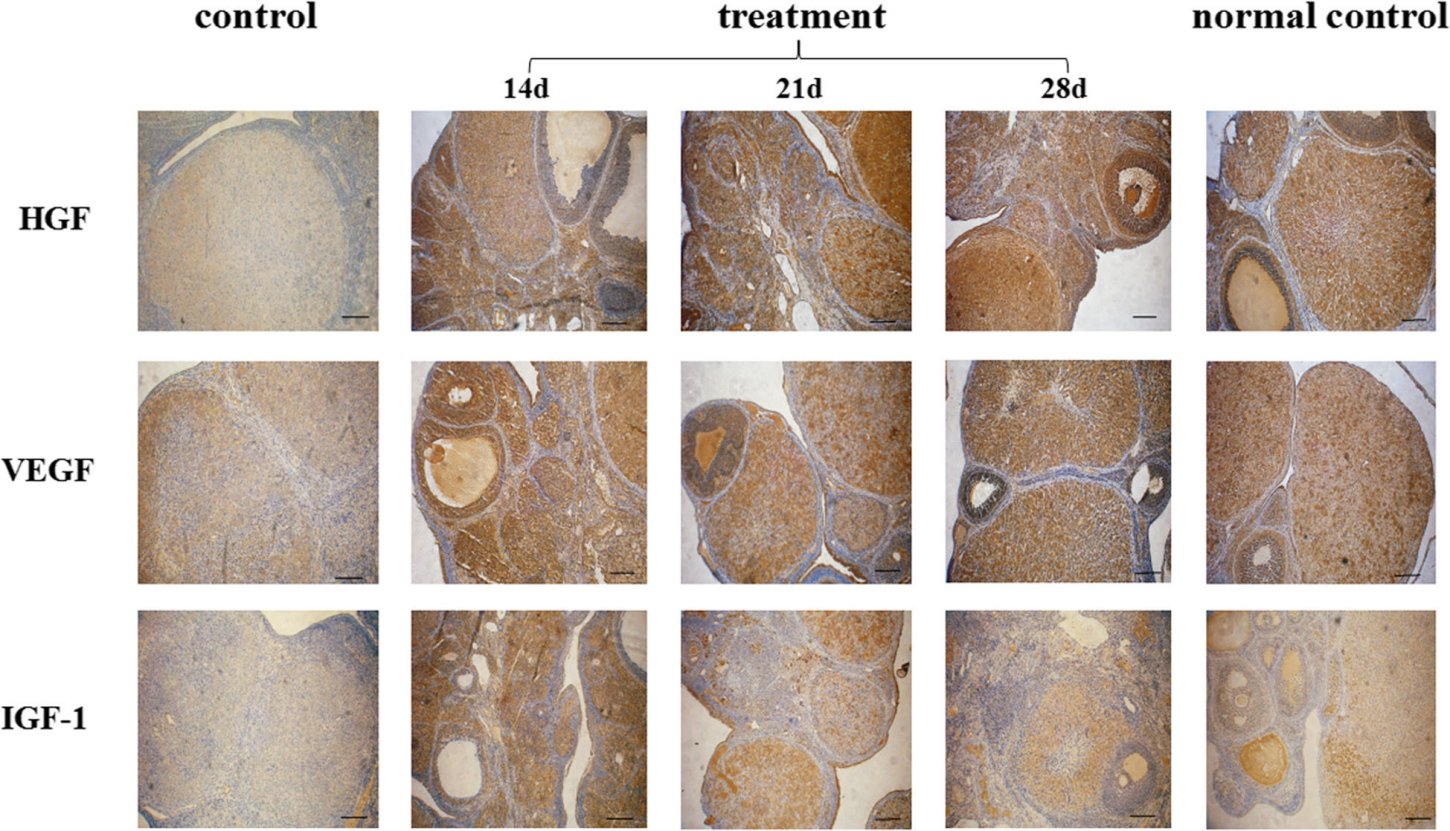
Immunohistochemistry (IHC) analysis for cytokines HGF, VEGF, and IGF-1 in ovaries after hUCMSCs transplantation. Images for control group treatment group and normal control group at three time points are listed. A *brown-yellow coloring* of the cytoplasm of the cells was deemed to be positive staining. It can be seen that treatment with hUCMSCs enhanced HGF, VEGF, and IGF-1 expression in ovaries. Scale bar = 200 um. *HGF* hepatocyte growth factor, *IGF-1* insulin-like growth factor-1, *VEGF* vascular endothelial cell growth factor

**Fig. 6:**
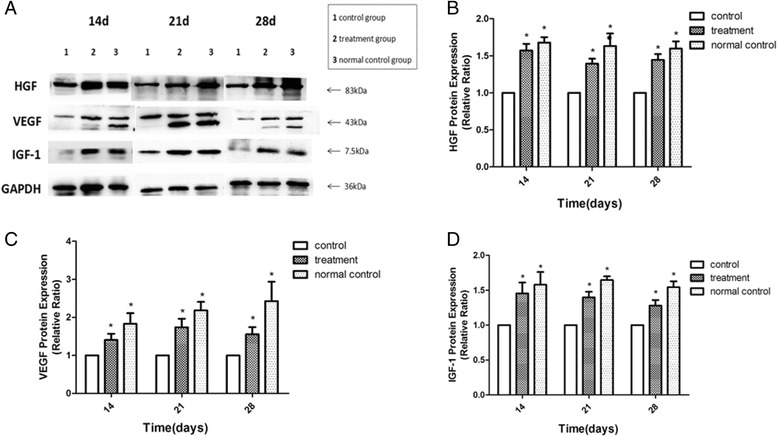
Images (**a**) and analysis of ovarian expression of HGF (**b**), VEGF (**c**), and IGF-1 (**d**) protein by western blot at different time points after hUCMSCs transplantation. GAPDH expression was used as internal reference. The protein expression in the control group was normalized as 1. Relative ratio of treatment group or normal control group to control group was viewed as the index of protein expression quantity. Data were shown in mean ± SD. ^*^ *P* < 0.05 vs. control group. *HGF* hepatocyte growth factor, *IGF-1* insulin-like growth factor-1, *VEGF* vascular endothelial cell growth factor

We found that HGF, VEGF, and IGF-1 were massively detected in rats’ ovarian granulosa cells, theca cells, and stromal cells through IHC. In addition, the expression of HGF, VEGF, and IGF-1 in the normal control group was frequently observed, while the control group showed a lower trend. After hUCMSCs transplantation, however, HGF, VEGF, and IGF-1 protein expression apparently rose compared to the control group (Fig. 5). The IRS of the three groups was statistically different (Tables 4, 5 and 6). This was later confirmed by western blot assay (Fig. 6). The standardization ratio of protein expression of HGF, VEGF, and IGF-1 in the treatment group and the normal control group significantly showed that ovarian expression of HGF, VEGF, and IGF-1 proteins of perimenopausal rats tended to be close to young rats after hUCMSCs treatment, compared to the control group.

**Table 4:** Comparison of the IHC scores for HGF

Group	14 d	21 d	28 d	*F* value	*P* value
Control	1.66 ± 0.29	1.66 ± 0.23	1.72 ± 0.33	0.073	0.930
Treatment	2.92 ± 0.53^*^	3.30 ± 0.42^*^	3.10 ± 0.22^*^	1.080	0.371
Normal control	3.04 ± 0.25^*^	3.12 ± 0.16^*^	3.12 ± 0.16^*^	0.274	0.765
F value	20.723	47.584	51.122		
*P* value	<0.001	<0.001	<0.001		

Data are shown as mean ± SD. (n = 5)

*IHC* immunohistochemistry, *HGF* hepatocyte growth factor

^*^ *P* < 0.05 vs. control group

**Table 5:** Comparison of the IHC scores for VEGF

Group	14 d	21 d	28 d	*F* value	*P* value
Control	1.68 ± 0.36	1.82 ± 0.46	1.54 ± 0.26	0.722	0.506
Treatment	2.90 ± 0.89^*^	2.78 ± 0.45^*^	3.02 ± 0.38^*^	0.190	0.829
Normal control	3.06 ± 0.62^*^	3.12 ± 0.54^*^	2.98 ± 0.66^*^	0.067	0.936
*F* value	6.574	9.657	16.482		
*P* value	0.012	0.003	<0.001		

Data are shown as mean ± SD. (n = 5)

*IHC* immunohistochemistry, *VEGF* vascular endothelial cell growth factor

^*^ *P* < 0.05 vs. control group

**Table 6:** Comparison of the IHC scores for IGF-1

group	14d	21d	28d	*F* value	*P* value
control	1.50 ± 0.12	1.30 ± 0.12	1.16 ± 0.21	6.00	0.016
treatment	2.20 ± 0.45^*^	2.12 ± 0.33^*^	2.10 ± 0.20^*^	0.118	0.890
Normal control	2.64 ± 0.30^*^	2.66 ± 0.28^*^	2.52 ± 0.24^*^	0.377	0.694
*F* value	15.840	34.312	51.957		
*P* value	<0.001	<0.001	<0.001		

Data are shown as mean ± SD. (n = 5)

*IHC* immunohistochemistry, *IGF-1* insulin-like growth factor-1

^*^ *P* < 0.05 vs. control group

## Discussion

While many researchers focus attention on the treatment of POF rats using adipose-derived mesenchymal stem cells (A-MSCs) [30], bone marrow mesenchymal stem cells (BMSCs) [31], and hUCMSCs [15, 16], our study focuses on the treatment of perimenopausal rats using hUCMSCs. In our study, the serum level of E_2_ and AMH decreased while FSH increased when the perimenopausal group of rats was selected. After hUCMSCs transplantation, we detected the serum index of E_2_, AMH, and FSH again and observed ovary tissue structures. The results suggested that hUCMSCs transplantation improved ovarian reserve function of perimenopausal rats. Although hUCMSCs have the ability to differentiate into germ cells, modern theories tend to deem [15, 16] that hUCMSCs do not change to oocyte-like structures or cells in vivo. Additionally, hUCMSCs labelled with fluorochrome mainly concentrated on the ovarian stroma, not on the ovarian follicle.

Follicle development needs the support of the vascular network in the ovary, hence inadequate ovarian stroma vessels may lead to a decline of oocyte quality with aging [32]. The development of vascular networks in the theca cell layer of the follicle is induced by angiogenic cytokines. In the ovary, the angiogenic factors produced by granulosa cells help to maintain the vasculature and health of the dominant follicles [33, 34]. A research study reported [35] that increasing age along with the reduction of ovarian stromal blood flow is a relatively late phenomenon, occurring only in women aged ≥41 years. As for angiogenic cytokines, VEGF is an effective mitogen for vascular endothelium [36] and it also stimulates vascular permeability [37]. Improving VEGF expression during the follicular stage may be helpful in increasing ovarian angiogenesis and the number of predominant follicles doomed for ovulation [38–41]. Beyond that, VEGF is a powerful survival factor for ovarian granulosa cell apoptosis and ovarian follicular atresia [42, 43]. Apart from VEGF, HGF is an important element of the internal follicular environment that accelerates the viability of growing follicles and enhances the proliferation of ovarian surface epithelium in order to replenish the area damaged due to expulsion of the ovum during ovulation [44, 45]. HGF, expressed both in thecal cells and granulosa cells of rat ovaries, may play its function as a modulator of the mesenchymal-epithelial cell reciprocities between theca cells and granulosa by facilitating cell proliferation and steroid hormone production [46]. A complete HGF system also supports granulosa cells growing via an anti-apoptotic effect [47]. Besides, as for other important cytokines in the ovary, IGF-1 is expressed in growing granulosa cells and healthy follicles, which cannot be detected in atretic follicles [48] and is necessary for the proliferation of granulosa cells at the early stage of folliculogenesis [49]. According to studies [50, 51], ovarian IGF-1 expression stimulates progesterone and estradiol production, and enhances granulosa cell FSH reactivity by improving FSH receptor expression. A study reported [52] that serum and follicular fluid levels of IGF-1 are decreased in reproductive aging women aged 40–45 years compared to young women aged 20–25 years. Thus, IGF-1 plays a crucial role in follicular development. In addition, a study suggested [16] that the amount of hUCMSCs in the rat ovarian tissue was basically constant without obvious proliferation for at least 8 weeks. This conforms to the results of our study; both serum hormone concentration and ovarian expression of HGF, VEGF, and IGF-1 protein remaining stable to 28 days after hUCMSCs transplantation.

hUCMSCs can secrete cytokines such as VEGF, HGF, and IGF-1. In addition, the ovarian expression of VEGF, HGF, and IGF-1 distinctly increased after hUCMSCs transplantation into perimenopausal rats via the tail vein. The results implied that hUCMSCs transplantation improved ovarian reserve function of perimenopausal rats through a paracrine mechanism.

## Conclusions

Through this study we suggest that hUCMSCs may be localized to the ovarian stroma and secreted cytokines after transplantation, affecting ovaries via a paracrine mechanism persisting at least 28 days. We can consider that hUCMSCs have therapeutic effects for perimenopausal rats to improve ovarian reserve function via a paracrine mechanism. However, based on these results, the time should be extended to more than 28 days to understand the timeliness of the therapeutic effects of hUCMSCs transplantation. Furthermore, whether the fertility of perimenopausal rats could be recovered after hUCMSCs transplantation needs further observation and testing. We hope this study can provide a theoretical foundation for perimenopausal treatment.

## Abbreviations

AMH: Anti-Müllerian hormone
A-MSCs: Adipose-derived mesenchymal stem cells
BMSCs: Bone marrow mesenchymal stem cells
E_2_: Estradiol
ELISA: Enzyme-linked immunosorbent assay
ESCs: Endometrial stromal cells
FSH: Follicle-stimulating hormone
H&E: hematoxylin and eosin
HGF: Hepatocyte growth factor
HRT: Hormone replacement therapy
hUCMSCs: Human umbilical cord mesenchymal stem cells
IGF-1: Insulin-like growth factor-1
IHC: Immunohistochemistry
IRS: Immunoreactive score
MSCs: human mesenchymal stem cells
NGF: Non-growing follicles
PBS: Phosphate-buffered saline
PCR: Polymerase chain reaction
POF: Premature ovarian failure
TGF-β: Transforming growth factor beta
VEGF: Vascular endothelial cell growth factor
WJ-MSCs: Human Wharton’s jelly mesenchymal stem cells
